# Depicting Changes in Tumor Biology in Response to Cetuximab Monotherapy or Combination Therapy by Apoptosis and Proliferation Imaging Using ^18^F-ICMT-11 and ^18^F-FLT PET

**DOI:** 10.2967/jnumed.118.209304

**Published:** 2018-10

**Authors:** Kathrin Heinzmann, Quang-Dé Nguyen, Davina Honess, Donna-Michelle Smith, Stephen Stribbling, Diana Brickute, Chris Barnes, John Griffiths, Eric Aboagye

**Affiliations:** 1 Department of Surgery and Cancer, Imperial College London, London, United Kingdom; and; 2 Cancer Research U.K. Cambridge Institute, Cambridge, United Kingdom

**Keywords:** apoptosis, proliferation, PET imaging, anti-EGFR-based therapy

## Abstract

Imaging biomarkers must demonstrate their value in monitoring treatment. Two PET tracers, the caspase-3/7–specific isatin-5-sulfonamide ^18^F-ICMT-11 (^18^F-(*S*)-1-((1-(2-fluoroethyl)-1H-[1,2,3]-triazol-4-yl)methyl)-5-(2(2,4-difluoro-phenoxymethyl)-pyrrolidine-1-sulfonyl)isatin) and ^18^F-FLT (3′-deoxy-3′-^18^F-fluorothymidine), were used to detect early treatment-induced changes in tumor biology and determine whether any of these changes indicate a response to cetuximab, administered as monotherapy or combination therapy with gemcitabine. **Methods:** In mice bearing cetuximab-sensitive H1975 tumors (non–small lung cancer), the effects of single or repeated doses of the antiepidermal growth factor receptor antibody cetuximab (10 mg/kg on day 1 only or on days 1 and 2) or a single dose of gemcitabine (125 mg/kg on day 2) were investigated by ^18^F-ICMT-11 or ^18^F-FLT on day 3. Imaging was also performed after 2 doses of cetuximab (days 1 and 2) in mice bearing cetuximab-insensitive HCT116 tumors (colorectal cancer). For imaging–histology comparison, tumors were evaluated for proliferation (Ki-67 and thymidine kinase 1 [TK1]), cell death (cleaved caspase-3 and terminal deoxynucleotidyl transferase–mediated deoxyuridine triphosphate nick-end labeling [TUNEL]), and target engagement (epidermal growth factor receptor expression) by immunohistochemistry, immunofluorescence, and immunoblotting, respectively. Tumor and plasma were analyzed for thymidine and gemcitabine metabolites by liquid chromatography–mass spectrometry. **Results:** Retention of both tracers was sensitive to cetuximab in H1975 tumors. ^18^F-ICMT-11 uptake and ex vivo cleaved caspase-3 staining notably increased in tumors treated with repeated doses of cetuximab (75%) and combination treatment (46%). Although a single dose of cetuximab was insufficient to induce apoptosis, it did affect proliferation. Significant reductions in tumor ^18^F-FLT uptake (44%–50%; *P* < 0.001) induced by cetuximab monotherapy and combination therapy were paralleled by a clear decrease in proliferation (Ki-67 decrease, 72%–95%; *P* < 0.0001), followed by a marked tumor growth delay. TK1 expression and tumor thymidine concentrations were profoundly reduced. Neither imaging tracer depicted the gemcitabine-induced tumor changes. However, cleaved caspase-3 and Ki-67 staining did not significantly differ after gemcitabine treatment whereas TK1 expression and thymidine concentrations increased. No cetuximab-induced modulation of the imaging tracers or other response markers was detected in the insensitive model of HCT116. **Conclusion:** ^18^F-ICMT-11 and ^18^F-FLT are valuable tools to assess cetuximab sensitivity depicting distinct and time-variant aspects of treatment response.

Two pivotal hallmarks of cancer are the capacity of cells to evade programmed cell death (apoptosis) and sustain deregulated proliferation (1). Thus, cancer drug development has predominantly focused on triggering apoptosis or halting cell proliferation, and noninvasive imaging tools demonstrating early efficacy for these agents singly or in combination are of great interest.

Our laboratory reported on the synthesis and application of a caspase-3/7–specific PET tracer, namely ^18^F-ICMT-11 (^18^F-(*S*)-1-((1-(2-fluoroethyl)-1H-[1,2,3]-triazol-4-yl)methyl)-5-(2(2,4-difluoro-phenoxymethyl)-pyrrolidine-1-sulfonyl)isatin) (2,3). When cells undergo apoptosis, caspases, a unique family of cysteine aspartate–specific proteases, are activated, acting as the main initiators and effectors. Activation of initiator caspases is triggered through death receptors such as Fas (extrinsic pathway) or release of cytochrome C from the mitochondria (intrinsic pathway), subsequently leading to activation of effector caspases such as caspase-3 and -7 by proteolytic processing into activated subunits. Once activated, effector caspases trigger cell destruction by breaking down cellular proteins and activating poly-adenosine diphosphate-ribose polymerase, an enzyme involved in DNA fragmentation.

Although it is important to monitor drug-induced apoptosis, most molecularly targeted therapies induce cytostasis; hence, it was of interest to compare ^18^F-ICMT-11 with a marker of cellular proliferation. The furthest progressed imaging tracer for proliferation is ^18^F-FLT (3′-deoxy-3′-^18^F-fluorothymidine) developed by Shields et al. (4). ^18^F-FLT is a thymidine analog that is taken up by the cell, phosphorylated by thymidine kinase 1 (TK1), but not incorporated into DNA to a notable extent. It is therefore primarily a marker of thymidine salvage pathway activity and has been correlated with standard markers of proliferation such as Ki-67 in some cancers (5).

Cetuximab (Erbitux; Merck Biopharma) is an example of a biologic agent used in current clinical practice. It is a chimeric human–murine monoclonal IgG1 antibody that blocks ligand binding to epidermal growth factor receptors (EGFRs), leading to a decrease in receptor dimerization, autophosphorylation, and receptor degradation. Cetuximab has demonstrated antitumor activity in a variety of models, including non–small cell lung cancer (NSCLC) models expressing wild-type and mutant EGFR (6), and has been reported to induce apoptosis in NSCLC model H1975 (7). NSCLC cells harboring somatic EGFR mutations, including the classic L858R mutation, are initially susceptible to tyrosine kinase inhibitors. However, acquired resistance ultimately ensues, commonly via a secondary T790M gatekeeper mutation. H1975 cells harbor both of these mutations characteristic of refractory NSCLC.

The combination of gemcitabine and cetuximab is reported to have higher efficacy than either drug alone (7), a finding that may be important for the management of patients who develop resistance to primary treatment. Cetuximab or gemcitabine treatment can singly reduce EGFR protein expression (8)—subsequent to phosphorylation priming the receptor for ubiquitination—leading to the hypothesis that combination of the two agents might optimize target inhibition.

Gemcitabine (2′,2′-difluorodeoxycytidine) is a chemotherapeutic agent used alone or in combination for the therapy of patients with NSCLC, breast cancer, ovarian cancer, and pancreatic cancer. Its mechanism of action is complex and still a topic of active research (9,10). It exerts its antiproliferative effect mostly through its two main metabolites, namely 2′,2′-difluorodeoxycytidine-5′-diphosphate and 2′,2′-difluorodeoxycytidine-5′-triphosphate, which inhibit DNA synthesis by reducing the deoxyribonucleoside triphosphate pool and chain termination. A third metabolite, 2′,2′-difluorodeoxyuridine, was shown to inhibit thymidylate synthase, the key enzyme of the thymidine de novo synthesis pathway (9), which is the alternative pathway to the thymidine salvage pathway. Modulation of ^18^F-FLT uptake, thymidine, and TK1 by gemcitabine was reported (11), making it an interesting candidate to investigate in combination therapy.

In the present study, the effects of cetuximab as monotherapy or in combination with chemotherapy, here gemcitabine, were investigated in the cetuximab-sensitive NSCLC cell line H1975. Tumor response was also evaluated in the human colorectal cancer model HCT116, which harbors a G13D mutation in *KRAS* rendering the tumor less sensitive to cetuximab treatment (12), as it is equally important to demonstrate that imaging biomarkers are negative when a tumor is nonresponsive to therapy.

## MATERIALS AND METHODS

### Animal Models and Cell Lines

BALB/c nude mice (Charles River Laboratories or Envigo) were inoculated in the dorsal neck region by subcutaneous injection with 1 × 10^6^ H1975 cells or 5 × 10^6^ HCT116 cells (both grown in RPMI-1640 medium supplemented with 10% fetal calf serum and glutamine; cell lines [ATCC] were used at a low passage number and routinely tested for mycoplasma)*.* All animal experiments were conducted in accordance with the U.K. Home Office guidance on the operation of “The Animals (Scientific Procedures) Act 1986 Amendment Regulations 2012” and with the National Cancer Research Institute guidelines for the welfare and use of animals in cancer research (13).

### In Vivo Drug Treatment and Imaging Schedule

H1975-bearing mice were treated with phosphate-buffered saline as vehicle or with a 10 mg/kg dose of cetuximab on day 1, a 10 mg/kg dose of cetuximab on days 1 and 2, a 125 mg/kg dose of gemcitabine (Sigma Aldrich) on day 2, or a combination of the 10 mg/kg dose of cetuximab on days 1 and 2 plus the 125 mg/kg dose of gemcitabine on day 2. Cetuximab was given by intravenous injection and gemcitabine by intraperitoneal injection. HCT116-bearing mice were treated with vehicle or the 2 daily doses of cetuximab only. The mice were imaged on day 3, or tumor volume was measured by calipers over 14 d (volume = (π/6)a × b × c).

### Ex Vivo Tumor Analysis

Tumor tissues were excised on day 3 after the start of treatment, fixed in formalin, embedded in paraffin, sectioned, and processed for Ki-67 (MIB-1 [DAKO]; 3,3′-diaminobenzidine detection [Invitrogen]), cleaved caspase-3 (D175) (catalog no. 9664; Cell Signaling Technologies), and terminal deoxynucleotidyl transferase-mediated deoxyuridine triphosphate nick-end labeling (TUNEL) (in situ cell death detection kit; Roche) assays or snap-frozen for further analysis. For fluorescence, 5 random fields per section were captured using an Olympus BX51 fluorescent microscope. For Ki-67 whole-mount slides were scanned. For immunoblotting, tumor tissue and cells were lysed in radioimmunoprecipitation assay buffer (Sigma, with Pierce protease and phosphatase inhibitor mini tablets [ThermoFisher], Precellys ceramic kits [1.4 mm; Bertin Technologies], and a Precellys 24 tissue homogenizer [Bertin Technologies]). Standard protocols were followed using 4%–15% Mini-PROTEAN TGX precast protein gels (BioRad); Trans-Blot Turbo nitrocellulose membranes (BioRad); primary antibodies to p1045 EGFR (catalog no. 2237; Cell Signaling Technologies), p1068 EGFR (catalog no. 3777; Cell Signaling Technologies), EGFR (catalog no. 4267; Cell Signaling Technologies), TK1 (ab76495; Abcam), thymidylate synthase (catalog no. 3766; Cell Signaling Technologies), thymidine phosphorylase (12383-1AP; Acris), equilibrative nucleoside transporter 1 (AB48607; Abcam), β-actin (ab6276; Abcam), and glyceraldehyde-3-phosphate dehydrogenase (catalog no. 5174; Cell Signaling Technologies); and secondary antibodies to sc-2004 and sc-2005 (Santa Cruz). For signal detection, GE Healthcare Amersham ECL Western blotting detection reagents and Hyperfilm ECL were used. ImageJ software (National Institutes of Health) was used for quantification.

### PET Imaging

^18^F-ICMT-11 was synthesized and radiolabeled according to previously described methodology (14). ^18^F-FLT was either commercially sourced (WBIC or PETNET) or produced in-house using a FASTlab cassette (GE Healthcare). Radiochemical purity was at least 96%.

The mice were anesthetized with isoflurane (∼2% in oxygen), and their body temperature was maintained at 37°C. The PET acquisition was performed in list mode on a GENISYS4 small-animal PET scanner (Sofie Biosciences), after the administration of about 1 MBq of ^18^F-ICMT-11 or ^18^F-FLT. Reconstruction was by 3-dimensional maximum-likelihood expectation maximization (60 iterations) (15). Uptake was calculated as percentage injected dose per milliliter (%ID/mL), evaluating peak (75th percentile), mean, and maximum tumor uptake relative to average radioactivity in the body for ^18^F-FLT PET at 50–60 min after injection. (Mean %ID/mL, maximum %ID/mL, and tumor-to-liver ratios are presented in the supplemental figures, available at http://jnm.snmjournals.org.) The mean of the summed ^18^F-ICMT-11 PET data at 40–60 min after injection was used for comparison, and the mean 95th percentile and maximum %ID/mL, calculated as previously described, are presented in the supplemental figures (3). Furthermore, all voxels and their associated intensities in each volume of interest were extracted and sorted as per their intensity frequency to give histograms sorting PET-based voxel intensity (3).

### Quantification of Thymidine and Gemcitabine Metabolites

Thymidine was analyzed as described previously (16), and gemcitabine metabolite analysis was performed according to Bapiro et al. (17). Briefly, the samples were prepared by homogenizing (tumor tissue) or mixing (plasma) with acetonitrile (50% v/v) and then centrifuging and evaporating the supernatant to dryness. The samples were then reconstituted in water before analysis by liquid chromatography–mass spectrometry. Sciex MultiQuant, version 2.1, was used for raw data integration and thymidine quantification. ThermoFisher Scientific LCquan, version 2.7, was used for quantification of gemcitabine and metabolites.

### Statistical Analysis

Results were expressed as mean ± SEM. The significance of multiple comparisons between datasets was determined using 1-way ANOVA with correction for multiple comparisons (Tukey) (comparing more than 2 conditions; H1975 tumors) or the unpaired Student *t* test with Welch correction (comparing 2 conditions; HCT116 tumors) unless otherwise stated.

## RESULTS

### Cetuximab as Monotherapy or in Combination with Gemcitabine Decreases EGFR Level in Cells

**FIGURE 1.**
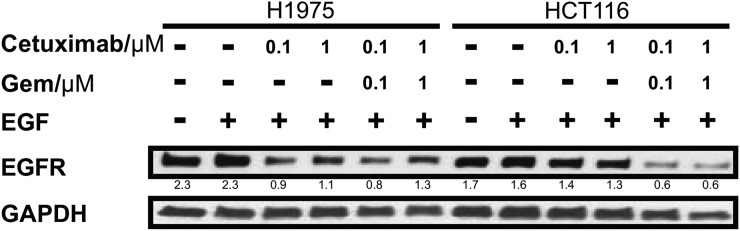
Receptor protein level decreases after cetuximab treatment in sensitive but not in insensitive model in vitro. H1975 and HCT116 cells were incubated with 1% serum-containing medium with vehicle (phosphate-buffered saline or cetuximab alone or with gemcitabine for 24 h at 0.1 or 1 μM. At 15 min before harvesting, EGF was added (final concentration, 10 ng/mL) as indicated (Supplemental Fig. 1). Protein expression was analyzed by immunoblotting. Signals were quantified, and ratios of receptor to GAPDH (glyceraldehyde-3-phosphate dehydrogenase; loading control) are noted between blots. Gem = gemcitabine.

To confirm the suitability of the two cell lines and to investigate whether combination treatment with gemcitabine causes a further decrease in EGFR protein expression, the response to cetuximab as a single agent and in combination with gemcitabine was characterized in H1975 and HCT116 cells (Fig. 1; Supplemental Fig. 1). The gemcitabine concentrations used were based on work by Feng et al. (8), and equimolar concentrations of cetuximab were used. EGFR protein expression levels decreased 3-fold when H1975 cells were treated with 0.1 μM cetuximab, whereas the effect of treatment was slightly attenuated at the higher dose (1 μM). The lack of a dose response suggests that inhibition of target reaches a plateau by 0.1 μM, although this inference was not further examined. In contrast, cetuximab only marginally affected EGFR levels in HCT116 cells. Interestingly, a clear but concentration-independent effect on receptor expression was observed in HCT116 cells after combination treatment.

### Cetuximab Increases ^18^F-ICMT-11 Retention After Repeated Dosing With or Without Gemcitabine

**FIGURE 2.**
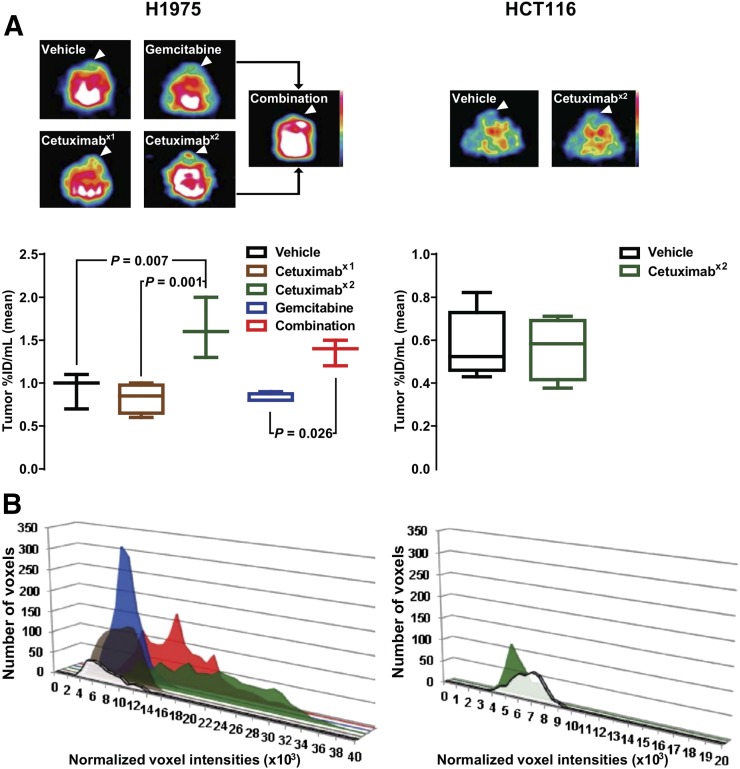
Repeated cetuximab dosing induces ^18^F-ICMT-11 retention. Mice bearing tumors (arrowheads) were treated with vehicle or with cetuximab on day 1 (x1), cetuximab on days 1 and 2 (x2), gemcitabine on day 2, or combination therapy and imaged with ^18^F-ICMT-11 PET on day 3. (A) Representative axial images and quantification (median and range). (B) Representative PET-based histograms sorting voxel intensity.

To investigate the effect of cetuximab-based treatment on ^18^F-ICMT-11 uptake, H1975-bearing mice were treated with vehicle, an intermediate dose of cetuximab either once (day 1) or twice (days 1 and 2), or gemcitabine (day 2) and then imaged on day 3 (Fig. 2; Supplemental Fig. 2). The drug concentrations were based on a study by Steiner et al. (7).

Although a single dose of cetuximab or gemcitabine alone did not induce any detectable change, there was a notable increase in tumor ^18^F-ICMT-11 intensity (vehicle: 0.93 ± 0.12 [*n* = 3]; cetuximab, 1 dose: 0.83 ± 0.09 %ID/mL [*n* = 4]; cetuximab, 2 doses: 1.63 ± 0.20 %ID/mL [*n* = 3]; gemcitabine: 0.83 ± 0.03 %ID/mL [*n* = 4]; combination: 1.37 ± 0.09 %ID/mL [*n* = 3]) (Fig. 2A) and a shift to a higher voxel intensity (Fig. 2B) as computed by PET-based voxel-intensity sorting in H1975 tumor–bearing mice after repeated dosing with cetuximab with and without gemcitabine. Only the repeated cetuximab dosing reached significance by PET analysis, compared with vehicle-treated tumors (*P* = 0.007). However, when studied ex vivo by γ-counting, both monotherapy and combination therapy showed highly significant effects on tracer retention (Supplemental Fig. 3; *P* < 0.0001 and *P* = 0.0007, respectively), but there was no difference between treated tumors with and without gemcitabine (*P* = 0.9).

Because the H1975 study indicated that gemcitabine did not markedly contribute to tracer retention, repeated dosing of cetuximab only was used to investigate whether a comparable change in retention could be observed in the cetuximab-insensitive model. None of the effects of treatment on imaging parameters, which had been modulated in the sensitive model, were detected in the HCT116 tumors (Fig. 2 [*P* = 0.9; *n* ≥ 4]; Supplemental Fig. 2).

**FIGURE 3.**
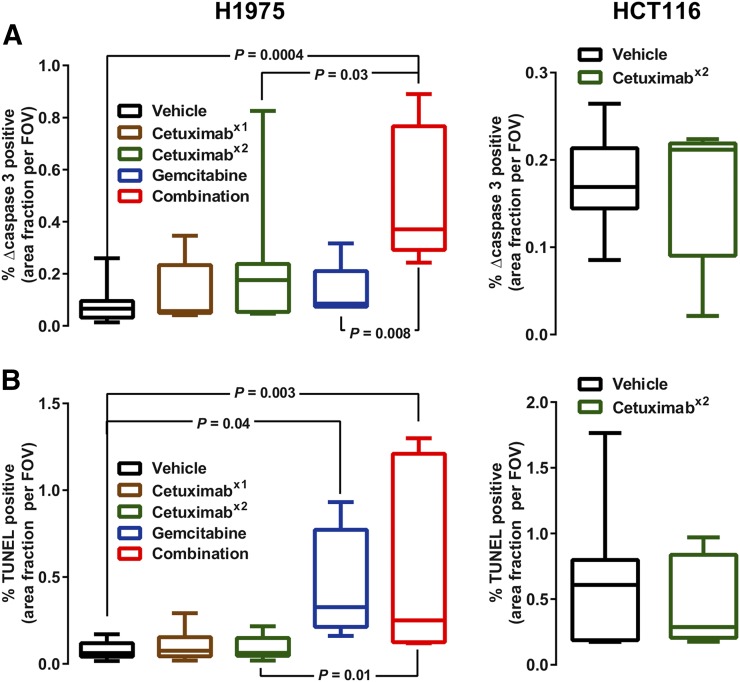
Ex vivo analysis shows selective increases in histologic apoptosis. Tumor sections were subjected to cleaved caspase-3 staining (A) and TUNEL assay (B). Quantification is shown as median and range. Representative images are shown in Supplemental Figure 4.

Immunofluorescence analysis of H1975 sections treated with a schedule identical to that of the imaging protocol (Fig. 3; Supplemental Fig. 4) revealed that staining of cleaved caspase-3 (Fig. 3A [*n* = 5–11]) had increased with the repeated dosing of cetuximab, and this change reached significance in tumors given the combination treatment (6.4-fold increase; *P* = 0.0004). An increase in TUNEL staining (Fig. 3B) was observed in the samples from H1975 tumors treated with gemcitabine independently of cetuximab treatment. There was no detectable change in either apoptosis marker in HCT116 tumors after treatment.

### Cetuximab Reduces Cell Proliferation as Measured by ^18^F-FLT PET and as Confirmed by Tumor Growth Study and Ki-67

**FIGURE 4.**
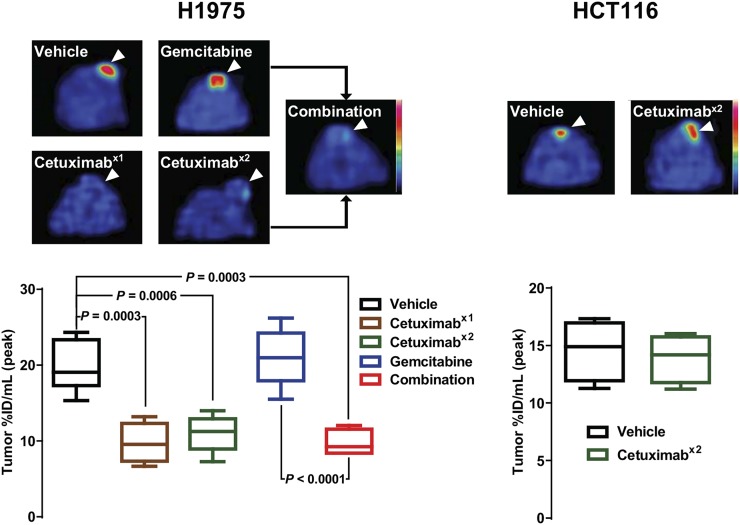
Cetuximab reduces ^18^F-FLT uptake. Mice bearing tumors (arrowheads) were treated with vehicle or with cetuximab on day 1 (x1), cetuximab on days 1 and 2 (x2), gemcitabine on day 2, or combination therapy and imaged with ^18^F-FLT PET on day 3. Representative axial images are shown. Quantification is shown as median and range.

To compare the performance of the apoptosis tracer with a measure of proliferation, ^18^F-FLT PET was used (Fig. 4; Supplemental Figs. 5 and 6).

The imaging analysis of H1975 tumor–bearing mice showed a distinct response to cetuximab treatment, with all cetuximab treatments leading to a significantly decreased peak uptake (vehicle: 19.7 ± 1.2 %ID/mL [*n* = 7]; cetuximab, 1 dose: 9.7 ± 1.3 %ID/mL [*n* = 4]; cetuximab, 2 doses: 11.0 ± 1.1 %ID/mL [*n* = 5]; combination: 9.7 ± 0.9 %ID/mL [*n* = 4]). Notably, no significant difference was observed between any of the cetuximab treatment groups. In contrast, gemcitabine-treated tumors showed uptake similar to the controls (21.0 ± 1.5 %ID/mL; *n* = 6).

^18^F-FLT PET imaging of HCT116 tumors showed no difference between the vehicle- and cetuximab-treated animals (vehicle: 14.6 ± 0.9 %ID/mL [*n* = 8]; cetuximab, 2 doses: 13.9 ± 1.0 %ID/mL [*n* = 4]; *P* > 0.05).

**FIGURE 5.**
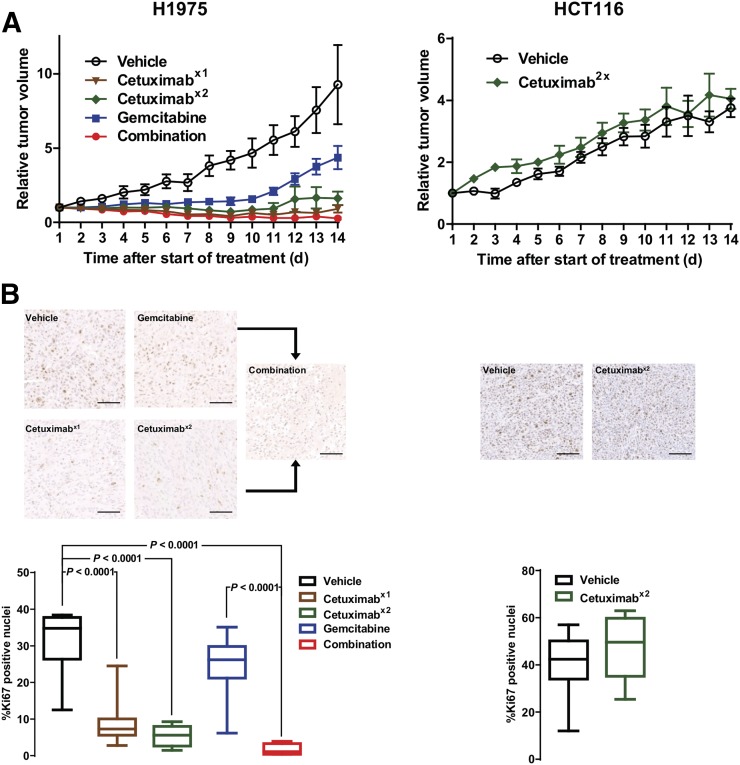
Cetuximab halts tumor growth and decreases Ki-67 expression in sensitive but not in insensitive tumors. Mice bearing tumors were treated with vehicle or with cetuximab on day 1 (x1), cetuximab on days 1 and 2 (x2), gemcitabine on day 2, or combination therapy. (A) Tumor volumes were determined and are expressed as fold change compared with volume at beginning of treatment (Supplemental Fig. 5). Statistical analysis was done by 2-way ANOVA with correction for multiple comparisons (Sidak). (B) Tumor sections treated as for growth study but collected on day 3 were stained for Ki-67 (bar = 100 μm). Representative images are shown. Quantification is shown as median and range.

Separate cohorts of H1975- and HCT116-bearing mice were subjected to the same treatment schedule as described for the imaging studies, and tumor volume was determined by caliper measurement for 14 d (Fig. 5A; Supplemental Fig. 7). In the H1975 model, tumor growth was sensitive to all treatments, and differences reached significance for single-dose cetuximab on day 5 (*P* = 0.03). For repeated cetuximab dosing, gemcitabine therapy, and combination therapy, the differences reached significance on day 6 after the start of treatment (*P* = 0.004, 0.01, and 0.0002, respectively). There was no difference between the group treated with cetuximab alone and the group treated with the combination, but tumors treated with gemcitabine alone started regrowing on day 11 (significantly different from tumors treated with the combination ; *P* = 0.005). In contrast, there was no difference between vehicle- and cetuximab-treated HCT116 tumors. Size changes in both tumor models agreed with the Ki-67 staining as measured on day 3 after the start of treatment (Fig. 5B), except for gemcitabine-treated H1975 tumors. In the sensitive model, Ki-67 was substantially reduced from 31.6% ± 2.7% to 9.0% ± 1.9% (1 dose of cetuximab), 5.4% ± 1.0% (2 doses of cetuximab), and 1.6% ± 0.8% (combination), whereas gemcitabine had no significant effect (*P* = 0.2).

### Cetuximab Modulates Target and Affects Key Factors of Nucleoside Metabolism

**FIGURE 6.**
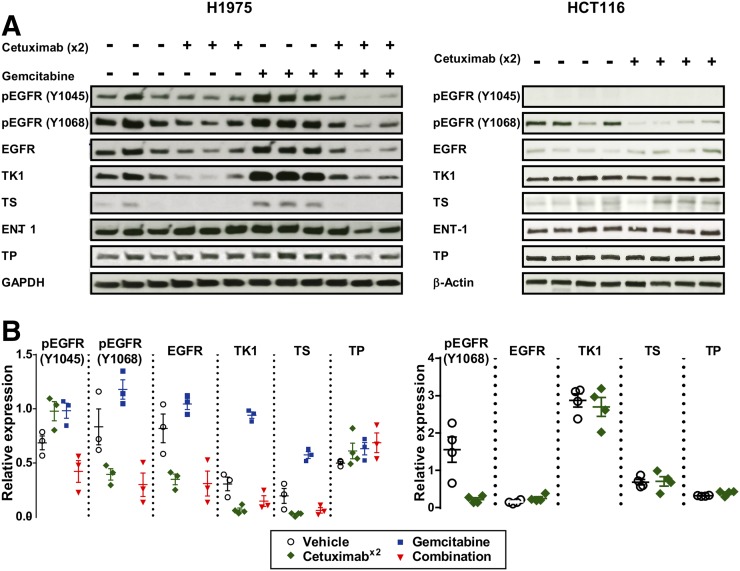
Cetuximab reduces expression of EGFR and proteins involved in thymidine metabolism. Mice bearing tumors were treated with vehicle or with cetuximab on day 1, cetuximab on days 1 and 2 (x2), gemcitabine on day 2, or combination therapy and collected on day 3. (A) Tumors were analyzed by immunoblotting against phosphorylated EGFR sites Y1045 (marking EGFR for ubiquitination) and Y1068 (marking receptor activity) or total expression of proteins as indicated. (B) Signals were quantified, and ratios of phosphorylated-to-total EGFR or protein-to-loading control (glyceraldehyde-3-phosphate dehydrogenase or β-actin) are shown. ENT 1 = equilibrative nucleoside transporter 1; GAPDH = glyceraldehyde-3-phosphate dehydrogenase; TP = thymidine phosphorylase; TS = thymidylate synthase.

Cetuximab treatment decreased EGFR phosphorylation at Y1068 and total protein concentration in H1975 tumors, contrasting with HCT116 tumors, in which lower phospho-Y1068 EGFR was seen but no change in total EGFR was detected (Fig. 6), confirming drug delivery and differential drug responses at a dose of 10 mg/kg in the two models. Interestingly, the ratio of phospho-Y1045 to EGFR increased when H1975 tumors were treated with cetuximab and gemcitabine only, suggesting that both treatments prime the receptor for degradation (18) in the combined treatment (marked reduction in total EGFR). Cetuximab treatment clearly reduced TK1 protein level (from 0.31 ± 0.06 to 0.06 ± 0.02), with the effect appearing less pronounced in the tumors treated in combination with gemcitabine (0.15 ± 0.05). In contrast, gemcitabine treatment increased TK1 protein level 3-fold (0.94 ± 0.03). Similar effects were observed for the expression of thymidylate synthase, whereas thymidine phosphorylase and equilibrative nucleoside transporter 1 protein levels appeared not to be affected by any treatment. Phospho-Y1045 EGFR was not detected in HCT116 tumors. Cetuximab treatment did not affect TK1, thymidylate synthase, thymidine phosphorylase, or equilibrative nucleoside transporter 1 protein expression level in HCT116 tumors.

### Tumor Thymidine Decreases After Cetuximab Treatment and Is Modulated by Gemcitabine

**FIGURE 7.**
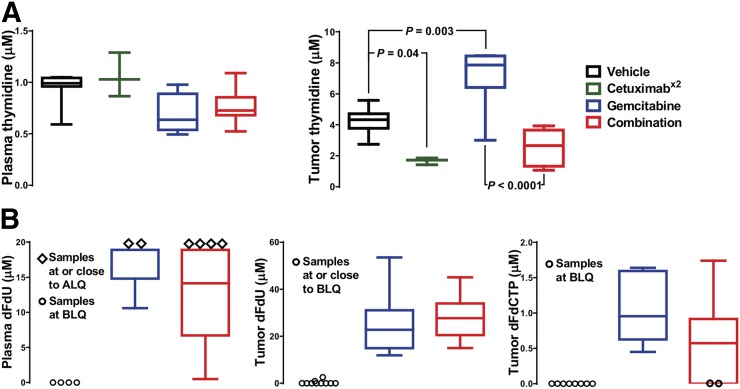
Endogenous thymidine metabolism is modulated by gemcitabine. Mice bearing H1975 tumors were treated with vehicle or with cetuximab on day 1, cetuximab on days 1 and 2 (x2), gemcitabine on day 2, or combination therapy, and thymidine (A) and gemcitabine (B) metabolite concentrations were measured in plasma and tumors on day 3. Quantification is shown as median and range. ALQ = above limit of quantitation; BLQ = below limit of quantification; dFdU = 2′,2′-difluorodeoxyuridine; dFdCTP = 2′,2′-difluorodeoxycytidine-5′-triphosphate.

As the treatment-modulated protein involved in thymidine nucleoside metabolism, endogenous thymidine was measured in tumor and plasma of H1975 tumor–bearing mice (Fig. 7A). Although cetuximab alone or in combination with gemcitabine had no effect on plasma thymidine concentrations, a small decrease in plasma thymidine concentrations (approaching significance, with *P* = 0.055) was observed after 24 h of gemcitabine treatment (*n* = 3–8), a finding that is similar to those of Schelhaas et al. (11). Cetuximab treatment significantly reduced tumor thymidine concentrations from 4.3 ± 0.3 μM (*n* = 8) to 1.7 ± 0.1 μM (*n* = 3). This effect appeared to be attenuated after combination treatment (*n* = 6; *P* = 0.1). In contrast, after gemcitabine treatment, tumor thymidine concentrations were markedly increased to 7.2 ± 0.9 μM (*n* = 6) in comparison to the controls (*P* = 0.003).

The following published evidence led us to measure gemcitabine metabolites in plasma and tumor (Fig. 7B): first, that the gemcitabine metabolite 2′,2′-difluorodeoxyuridine can inhibit thymidylate synthase (9), and second, that the gemcitabine metabolites 2′,2′-difluorodeoxycytidine and 2′,2′-difluorodeoxyuridine can compete with ^18^F-FLT by sharing some nucleoside metabolic enzymes and transporters (11). There was no detectable 2′,2′-difluorodeoxycytidine in the plasma, but 2′,2′-difluorodeoxyuridine was present 24 h after treatment with gemcitabine and combination therapy. 2′,2′-difluorodeoxycytidine-5′-triphosphate is not stable in plasma and was therefore not assayed. However, both 2′,2′-difluorodeoxyuridine and 2′,2′-difluorodeoxycytidine-5′-triphosphate were detected in the tumors treated with gemcitabine or the combination. No statistical difference was found between monotherapy and combination therapy.

## DISCUSSION

The main aim of this study was to investigate which aspects of treatment response or the lack of such can be detected early by ^18^F-ICMT-11 or ^18^F-FLT PET. We demonstrated that the two tracers depict complementary aspects of tumor biology early after treatment, namely the balance between apoptosis and proliferation, which varies according to the treatment.

Differences in molecular pharmacology, detected as cognate changes in caspase-3/7 imaging biomarker output, were observed in this study. Regarding target engagement, the higher dose of cetuximab after repeated dosing than after a single dose in the H1975 tumors, the lack of EGFR protein degradation sustaining proliferative signaling in the less sensitive HCT116 tumors, and the similar lack of EGFR protein degradation in the H1975 model after gemcitabine monotherapy could explain the selective increase in tumor ^18^F-ICMT-11 uptake observed in the H1975 model after repeated dosing and combination therapy (Fig. 2; Supplemental Figs. 2 and 3). Changes in ^18^F-ICMT-11 uptake were broadly in agreement with the ex vivo cleaved caspase-3 staining (Fig. 3). Changes in cleaved caspase-3 were small and positivity was heterogeneous across the tumor slices. Thus, representative quantification and correlation between histology and whole-tumor imaging is challenging, especially because apoptotic events appeared to be localized. However, consideration of the two measurements of apoptosis and visual assessment suggests that a considerable number of apoptotic events are required for reliable detection by ^18^F-ICMT-11 imaging; cleaved caspase-7, also detected by ^18^F-ICMT-11, was not measured. Interestingly, a profound increase in TUNEL staining was observed in the samples from H1975 tumors treated with combination therapy or a single dose of gemcitabine, suggesting that gemcitabine does induce apoptosis but that at 24 h after treatment, biologic effects are not detected by ^18^F-ICMT-11 because activation of caspase-3 is an early and highly dynamic apoptosis event (3). Of note, Schelhaas et al., using a lower dose of gemcitabine (100 mg/kg), did not observe apoptosis at 24 h in the H1975 model and did not investigate earlier time points (11).

Although complex, the ^18^F-FLT imaging results were broadly in agreement with measurements of tumor growth delay (Fig. 5A) and with target engagement as seen by immunoblotting (Fig. 6). Analysis of Ki-67 staining (Fig. 5B) demonstrated that proliferation had halted by day 3 after the start of cetuximab monotherapy in the drug-sensitive model, with a concomitant reduction in TK1 and tumor thymidine (Figs. 6 and 7A). Despite some mechanistic changes, the combination of cetuximab with gemcitabine had little effect on ^18^F-FLT uptake, Ki-67, EGFR protein level, or tumor growth delay in comparison to cetuximab monotherapy, suggesting that there is little benefit to adding gemcitabine to the treatment regime when a highly effective dose of cetuximab is used. However, long-term studies, which were beyond the scope of this work, would be required to investigate whether outcome differs at a later time point. Regarding gemcitabine administered as monotherapy, Schelhaas et al. had observed a decrease in ^18^F-FLT retention after 24 h of gemcitabine treatment at 100 mg/kg in H1975 tumors (11), whereas there was no significant difference in our study using a higher dose of gemcitabine. In comparison with other gemcitabine-induced changes, gemcitabine did have a small but insignificant effect on plasma thymidine in our study whereas the marked effect on tumor thymidine was comparable but marginally more pronounced in the study by Schelhaas et al. This difference could be due either to differences such as drug formulation or to the mouse strain, potentially affecting drug metabolism. Because no metabolites were studied by Schelhaas et al. at that time point, the reason for the difference remains speculative. However, in our study, the presence of two gemcitabine metabolites (Fig. 7B) that could have opposing effects on ^18^F-FLT uptake (inhibition of DNA synthesis and thymidine salvage pathway upregulation by thymidylate synthase inhibition (19)) indicates that at 24 h after treatment an equilibrium is reached and, thus, that there is no net change in tracer uptake.

Although gemcitabine affected thymidine metabolism, ^18^F-FLT retention was not appreciably altered. Concerning other potentially confounding factors, cetuximab has been reported to cause immune cell infiltration (20), but neither ^18^F-ICMT-11 (Supplemental Fig. 8 (21,22)) nor ^18^F-FLT (23) showed notable changes in uptake in preclinical models of inflammation, although such effects are likely attenuated in the immunocompromised mice used in our studies. Concerning biomarker specificity, the main focus of our study, the markedly modulated aspects of treatment response that were detected by the PET tracers in the cetuximab-sensitive model were also investigated in the insensitive HCT116 tumor model and found to be specific to treatment response.

## CONCLUSION

This study showed that despite the complications in interpretation of the imaging output by addition of gemcitabine, changes in the uptake of ^18^F-ICMT-11 and ^18^F-FLT are useful biomarkers of response to treatments containing cetuximab, demonstrating that the balance between proliferation and apoptosis is altered in relation to drug schedule, and providing a rationale for their use in future human studies. The study exemplifies how heterogeneous (spatially and temporally) tumors react to treatment and underscores the importance of multiplexing imaging readouts (e.g., by generating a ratio image) to better characterize therapy-induced modulation of tumor biology.

## DISCLOSURE

This work was supported by EU FP7 IMI project QuIC-ConCePT (grant 115151; financial contributions from the European Union’s Seventh Framework Program [FP7/2007-2013] and EFPIA companies’ in-kind contribution) and CR-UK grant C2536/A16584. No other potential conflict of interest relevant to this article was reported.
