# Supplementary material for: Depicting Changes in Tumor Biology in Response to Cetuximab Monotherapy or Combination Therapy by Apoptosis and Proliferation Imaging Using 18F-ICMT-11 and 18F-FLT PET
Source: J Nucl Med. 2018 Oct;59(10):1558–65. doi: 10.2967/jnumed.118.209304 (PMC6167530; doi:10.2967/jnumed.118.209304)
Supplement: Supplementary file 1 [file jnm209304SupplementalData.pdf]

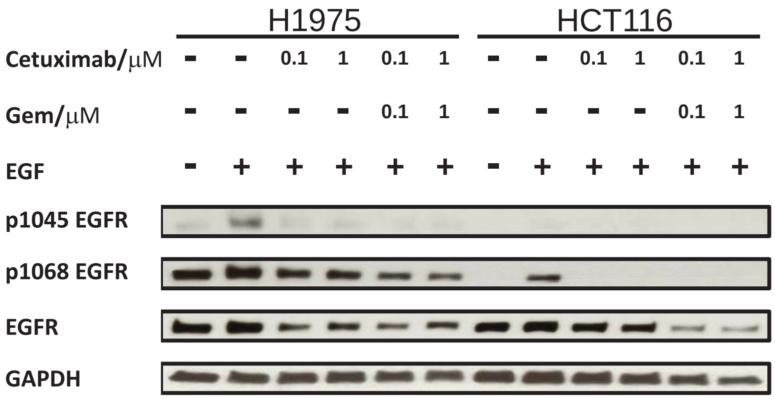

SUPPLEMENTAL FIGURE 1. Cetuximab affects receptor phosphorylation and protein level *in vitro*. H1975 and HCT116 cells were incubated with 1% serum-containing media with vehicle (phosphate buffered saline) or cetuximab alone or with gemcitabine ('Gem') for 24h at 0.1 or 1μM. 15min before harvesting, EGF was added (final concentration 10ng/mL) as indicated to confirm specific stimulation by phosphorylation and its inhibition by cetuximab. Tumors were analyzed by immunoblot against the phosphorylated EGFR sites Y1045 (marking EGFR for ubiquitination) and Y1068 (marking receptor activity) or total expression of proteins as indicated. GAPDH was used as a loading control.

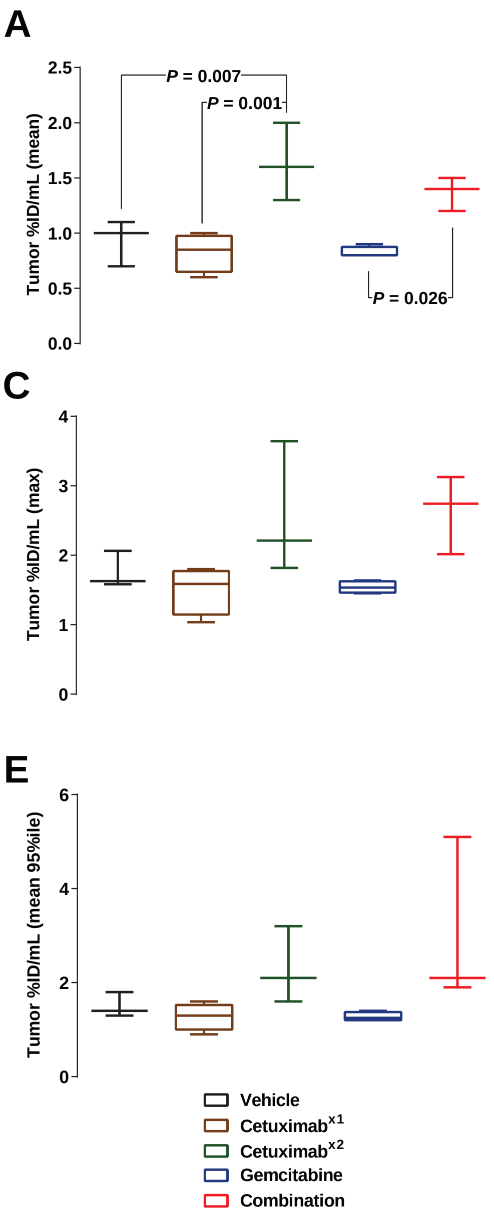

SUPPLEMENTAL FIGURE 2. Cetuximab selectively induces ^{18}F-ICMT-11 uptake (complementing Figure 2). H1975 tumor-bearing mice were treated with vehicle, one (day 1) or two doses (day 1 and day 2) of cetuximab (‘Cetuximab^{x1}’ or ‘Cetuximab^{x2}’) and/or gemcitabine (one dose (day 2) ‘Gemcitabine’ or after repeat-dosing of cetuximab ‘Combination’). HCT116 tumor-bearing mice were treated with vehicle or two doses of cetuximab (day 1 and day 2; ‘Cetuximab^{x2}’). %ID/mL (mean (reproduced for easy comparison; A (H1975) and B (HCT116)), maximum (C (H1975) and D (HCT116)) and mean 95% (average of the top 5%; E (H1975) and F (HCT116)) are shown. Results are represented by box-and-whisker plots with minima and maxima.

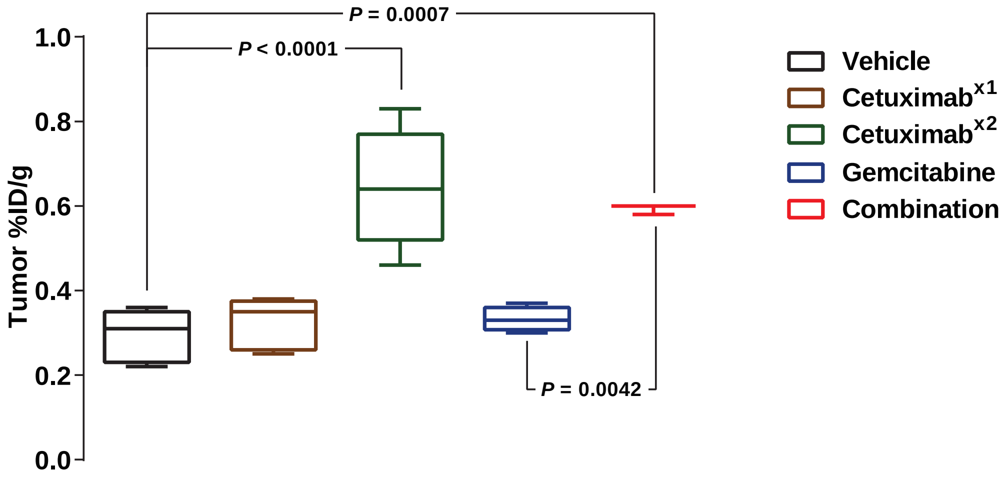

SUPPLEMENTAL FIGURE 3. Cetuximab dosing induces ^{18}F-ICMT-11 retention assessed by tissue excision and γ-counting. H1975 tumor-bearing mice were treated with vehicle (n=5), one (day 1; n=5) or two doses (day 1 and day 2; n=5) of cetuximab (‘Cetuximab^{x1}’ or ‘Cetuximab^{x2}’) and/or gemcitabine (one dose on day 2 (‘Gemcitabine’ (n=4)) or after repeat-dosing of cetuximab (‘Combination’ (n=3)). Mice were injected with ^{18}F-ICMT-11 PET (day 3) and after 1h tumors were excised and radioactivity counted. Results are represented by box-and-whisker plots with minima and maxima.

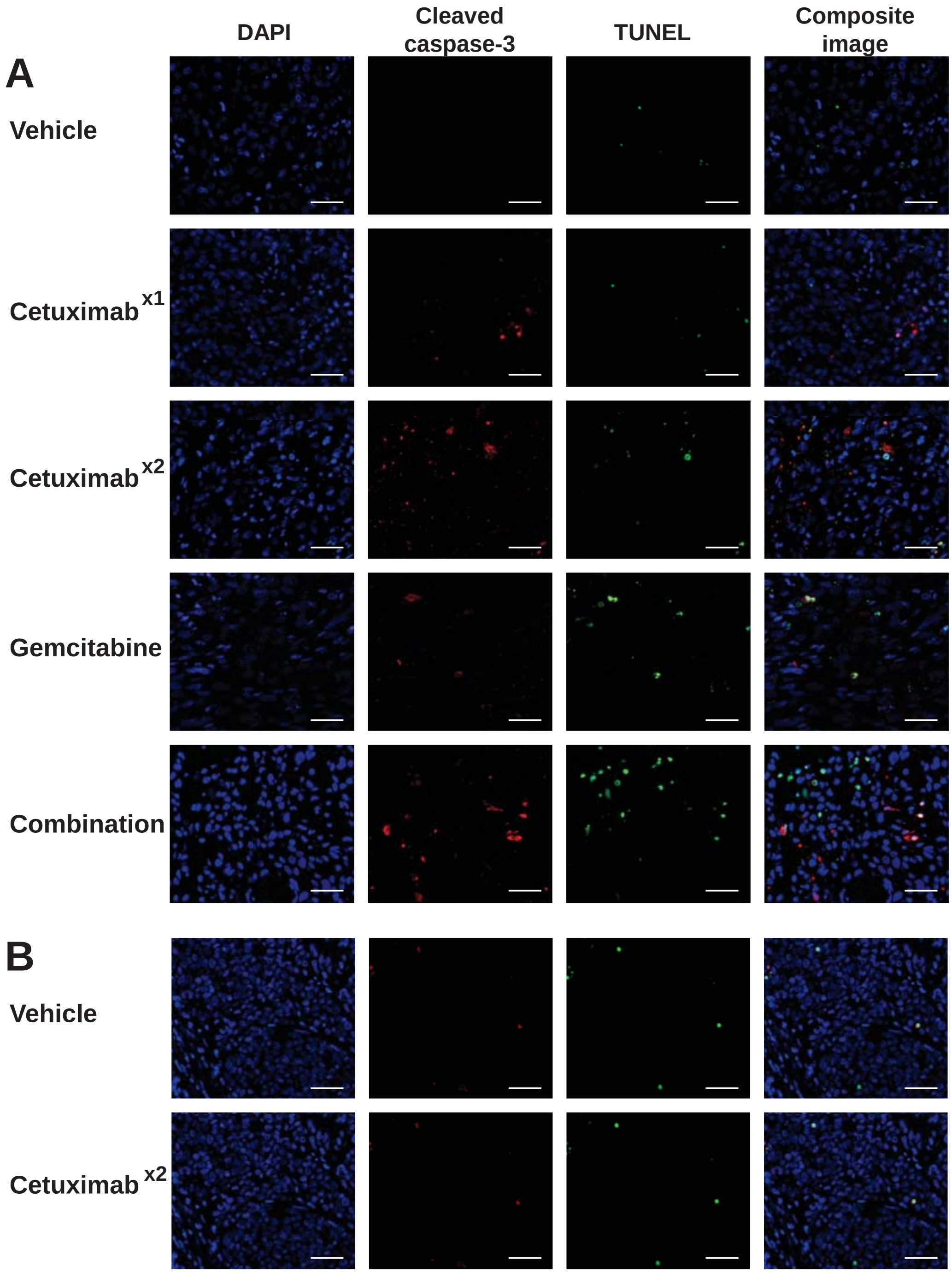

SUPPLEMENTAL FIGURE 4. *Ex vivo* analysis of tumor tissue by cleaved caspase-3 staining and TUNEL (complementing Figure 3). Tumor tissues were excised, fixed in formalin, embedded in paraffin, sectioned, and processed for fluorescent detection of active caspase-3 and DNA degradation terminal deoxynucleotidyl transferase–mediated dUTP nick end labeling (TUNEL) using the cleaved caspase-3 monoclonal antibody (D175; CST; #9664) coupled with Alexa Fluor 594 goat anti-rabbit red; Invitrogen; (#A-11037) and the In Situ Cell Death Detection Kit (Roche), respectively. The ProLong Gold Antifade mounting solution (Invitrogen) containing 4′,6-diamidino-2-phenylindole (DAPI) was added to tissue sections prior to coverslip mounting. All assays were conducted according to the manufacturer's instructions. Each stain is shown separately and as composite image for H1975 tumors (A) and HCT116 (B). Magnification 200x; white bar = 50μm.

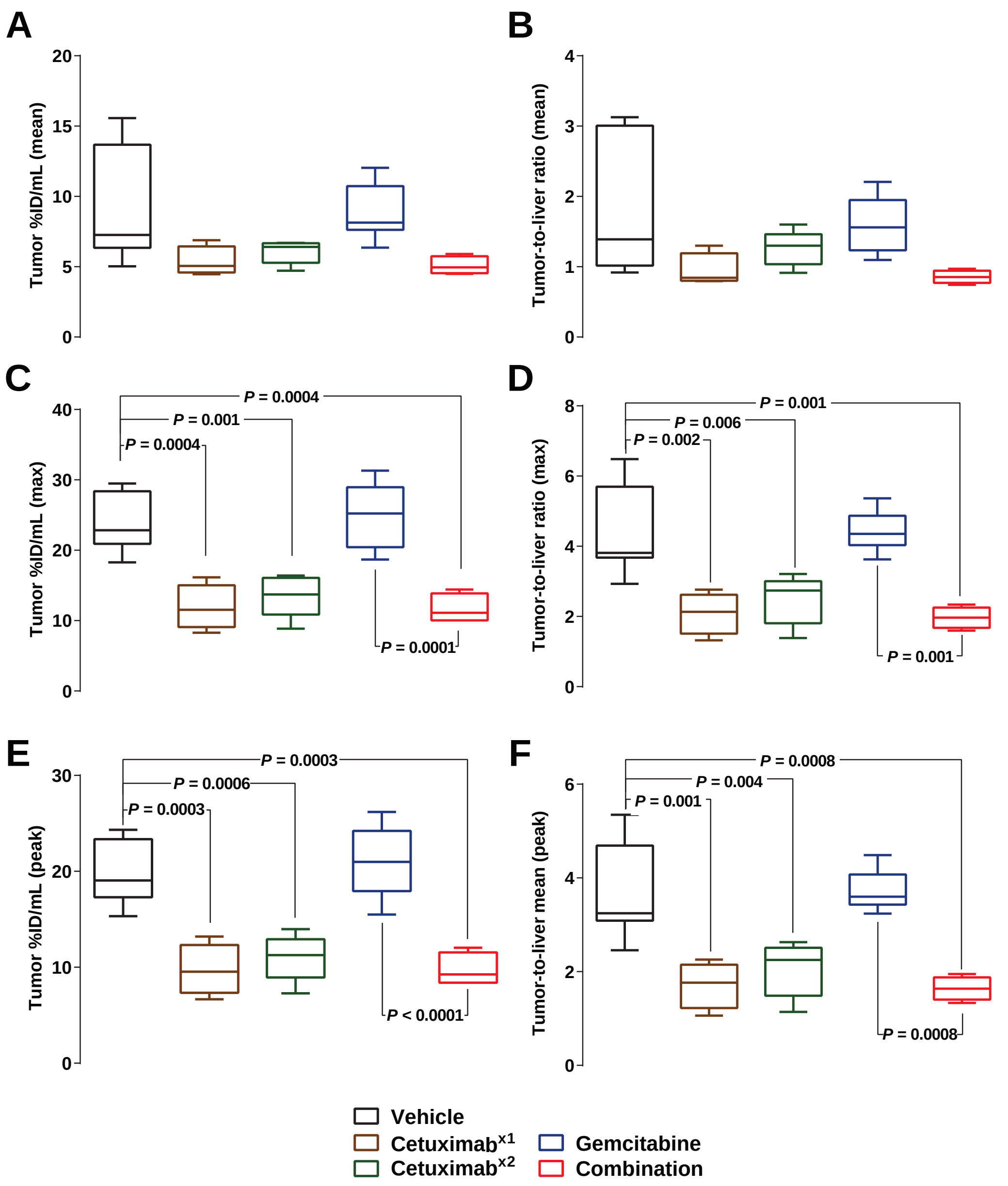

SUPPLEMENTAL FIGURE 5. Cetuximab reduces ^{18}F-FLT uptake in H1975 tumors (complementing Figure 4). H1975 tumor-bearing mice were treated with vehicle, one (day 1) or two doses (day 1 and day 2) of cetuximab ('Cetuximab^{x1}' or 'Cetuximab^{x2}') and/or gemcitabine (one dose (day 2) 'Gemcitabine' or after repeat-dosing of cetuximab 'Combination'). %ID/mL (mean (A), max (C) and peak (reproduced for easy comparison; 75%ile; E), and tumor-to-liver ratio (mean (B), max (D) and peak (75%ile; F) are shown. Results are represented by box-and-whisker plots with minima and maxima.

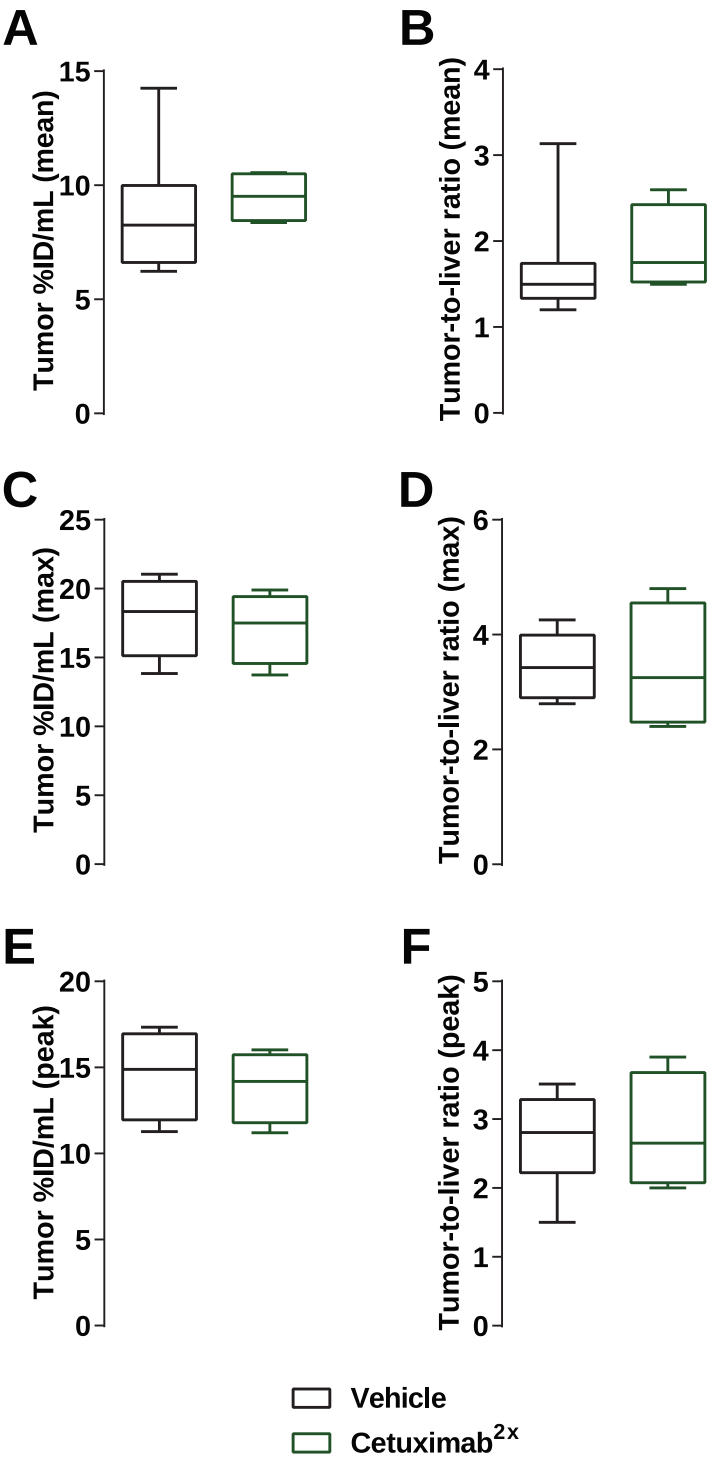

SUPPLEMENTAL FIGURE 6. Cetuximab does not reduce ^{18}F-FLT uptake in HCT116 tumors (complementing Figure 4). HCT116 tumor-bearing mice were treated with vehicle or two doses of cetuximab (day 1 and day 2; 'Cetuximab^{x2}'). %ID/mL (mean (A), max (C) and peak (reproduced for easy comparison; 75%ile; E), and tumor-to-liver ratio (mean (B), max (D) and peak (75%ile; F) are shown. Results are represented by box-and-whisker plots with minima and maxima.

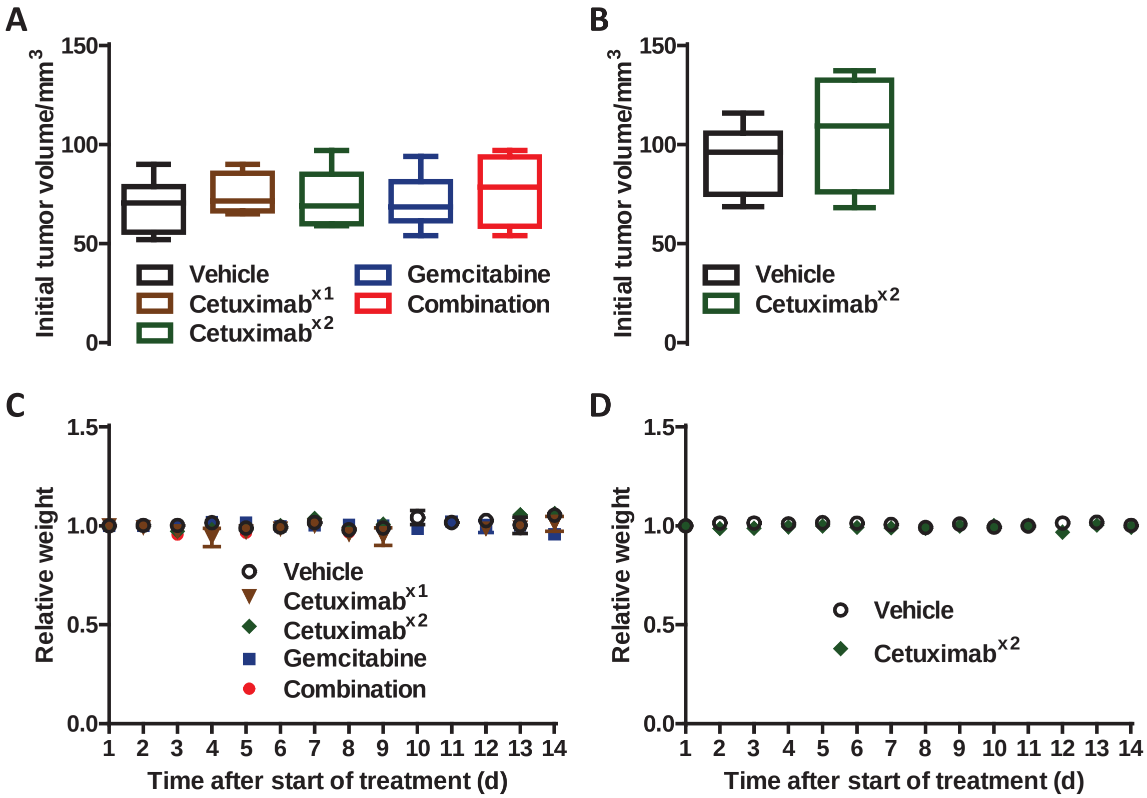

SUPPLEMENTAL FIGURE 7. Supplemental material to tumor growth studies (complementing Figure 5). The distribution of tumor volumes (determined by caliper measurement) at the start of treatment is shown for H1975 (A) and HCT116 (B) tumors. Data is presented as box-and-whisker plots with minima and maxima. Numbers were as follows: H1975 (Vehicle (n=6); Cetuximab^{1x} (n=6); Cetuximab^{x2} (n=5); Gemcitabine (n=3); Combination (n=4)) and HCT116 (Vehicle (n=8); Cetuximab^{x2} (n=4)). H1975 tumor-bearing mice were then treated with vehicle, one (day 1) or two doses (day 1 and day 2) of cetuximab ('Cetuximab^{x1}' or 'Cetuximab^{x2}') and/or gemcitabine (one dose on day 2 ('Combination' or 'Gemcitabine'). HCT116 tumor-bearing mice were treated with vehicle or two doses of cetuximab (day 1 and day 2; 'Cetuximab^{x2}'). Over the course of the study, mouse body weight was monitored and the measurements tumor-bearing mice are presented for H1975 tumors in C and for HCT116 tumors in D.

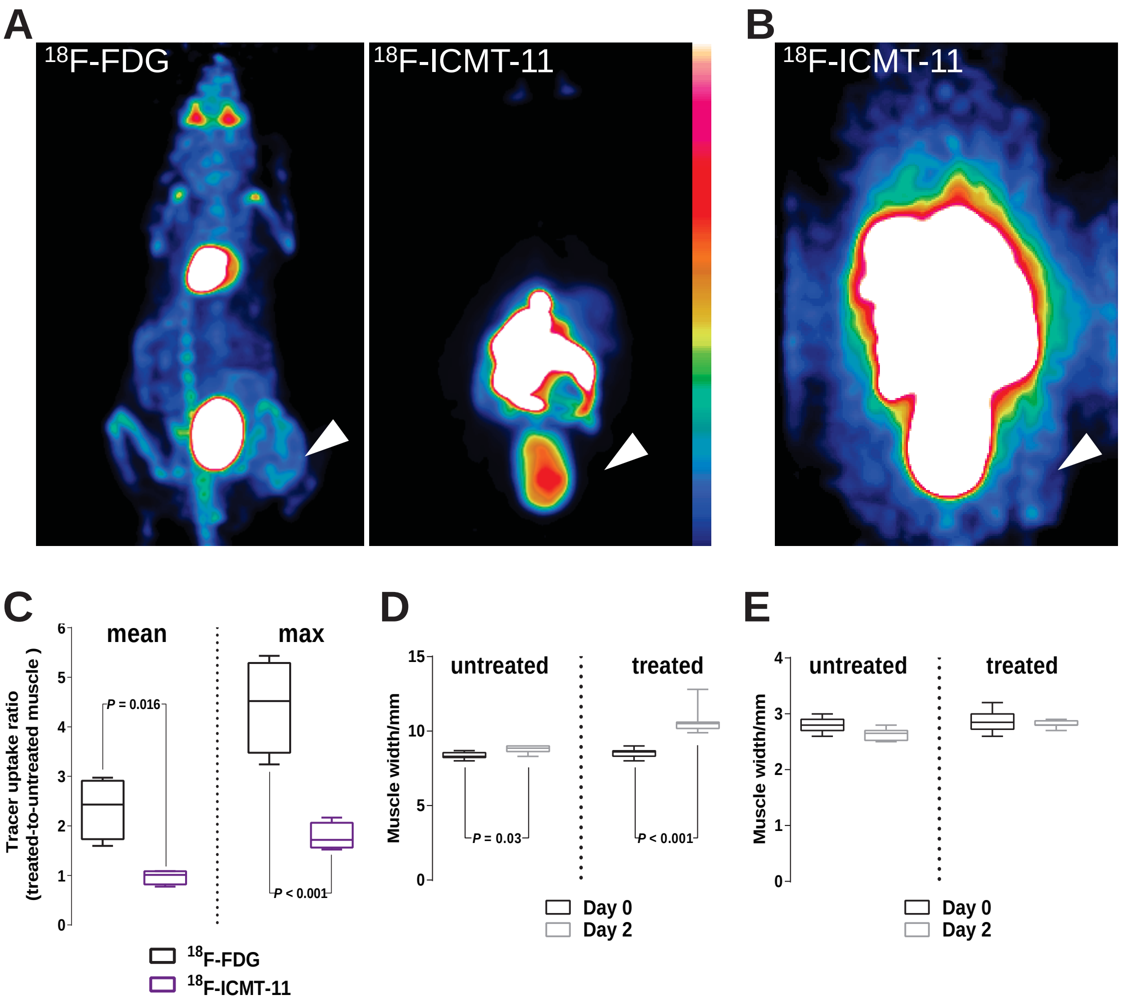

SUPPLEMENTAL FIGURE 8. ^{18}F-ICMT is not taken up in an aseptic inflammation model. BALB/c wildtype mice were injected intramuscularly (posterior thigh muscle) with turpentine oil, using a method adapted from van Waarde et al (*1*) and previously described by our lab (*2,3*). 48h after the injection, the mice were injected intravenously with ^{18}F-FDG (n=4) and ^{18}F-ICMT-11 (n=4) and imaged. Representative coronal images are shown in A (Maximum Intensity Projections) and B (^{18}F-ICMT-11 PET image to show muscle). White arrowheads indicate inflamed muscle. Results of the imaging are presented (C, 40-60min after tracer injection) as ratios of treated-to-untreated muscle and the muscle width of treated and untreated muscle, and control muscle (calf) are shown in D and E. Results of the quantification are represented by box-and-whisker plots with minima and maxima. The data show that compared to ^{18}F-ICMT-11 uptake, the uptake of ^{18}F-FDG is profoundly higher in the inflamed muscle. The turpentine oil-treated muscles are comparatively bigger than the untreated muscles showing the inflammation. Interestingly also the untreated thigh muscles are significantly bigger but not to the same extent. It is possible that because the mice favor the non-inflamed leg, the muscle size increased slightly. ^{18}F-FDG was purchased from PETNET (Northwood UK, Radiochemical purity: 99.95%).

1. van Waarde A, Cobben DC, Suurmeijer AJ, et al. Selectivity of 18F-FLT and 18F-FDG for differentiating tumor from inflammation in a rodent model. *J Nucl Med.* 2004;45:695-700.

2. Witney TH, Pisaneschi F, Alam IS, et al. Preclinical evaluation of 3-18F-fluoro-2,2-dimethylpropionic acid as an imaging agent for tumor detection. *J Nucl Med.* 2014;55:1506-1512.

3. Witney TH, Carroll L, Alam IS, et al. A novel radiotracer to image glycogen metabolism in tumors by positron emission tomography. *Cancer Res.* 2014;74:1319-1328.
